# An exploration of the enablers and barriers in access to the Dutch healthcare system among Ghanaians in Amsterdam

**DOI:** 10.1186/1472-6963-12-75

**Published:** 2012-03-24

**Authors:** Linda Boateng, Mary Nicolaou, Henriëtte Dijkshoorn, Karien Stronks, Charles Agyemang

**Affiliations:** 1 Department of Public Health, Academic Medical Centre, University of Amsterdam, Postbus 22660, 1100DD Amsterdam, The Netherlands; 2 Municipal Health Service, Amsterdam, The Netherlands

**Keywords:** Access to healthcare, Perceptions of healthcare, Ethnicity, African

## Abstract

**Background:**

Sub-Saharan African populations are growing in many European countries. Data on the health of these populations are rare. Additionally, many sub-Saharan African migrants are confronted with issues of low socio-economic status, acculturation and language difficulties, which may hamper their access to health care. Despite the identification of some of those barriers, little is known about the enabling factors. Knowledge about the enablers and barriers in access to healthcare experienced is important in addressing their health needs and promoting healthcare access. This study aimed to investigate the enabling factors as well as barriers in access to the Dutch healthcare system among the largest sub-Saharan African migrant group (Ghanaians) living in Amsterdam, the Netherlands.

**Methods:**

Six focus groups were conducted from November 2009 to February 2010. A semi-structured interview guideline was used. Discussions were conducted in English or Twi (Ghanaian dialect), recorded and transcribed verbatim. Analysis was based on the Andersen model of healthcare utilisation using MAXQDA software.

**Results:**

Knowledge and perceived quality of the health system, awareness of diseases, family and community support, community initiatives and availability of social support were the main enablers to the healthcare system. Difficulties with the Dutch language and mistrust in health care providers were major barriers in access to healthcare.

**Conclusions:**

Access to healthcare is facilitated mainly by knowledge of and the perceived efficiency and quality of the Dutch healthcare system. However, poor Dutch language proficiency and mistrust in health care providers appear to be important barriers in accessing healthcare. The enablers and barriers identified by this study provide useful information for promoting healthcare access among this and similar Sub-Saharan African communities.

## Background

Generally, migrant and ethnic minority populations perceive worse health than majority host populations [1,2]. Community perceptions of heath, such as cultural health beliefs and differences in understanding of diseases, have been linked to poor health care access [3-5]. However, little is known about the enablers and barriers in access to healthcare experienced in migrant and ethnic minority groups, even less so among recent sub-Saharan African populations [2,6]. There is also evidence that many ethnic minority groups are often dissatisfied with the host country's healthcare system due mainly to language and cultural barriers which affect their quality of healthcare [5,7]. The Global Consultation on Migrant Health has identified these barriers as contributing to limiting migrants' access to healthcare [8]. The growing numbers of ethnic minorities in Europe demands an understanding of their health status and needs to foster equity and integration [1].

Migrants from sub-Saharan Africa are one of the fastest growing migrant populations in Europe. The Organization for Economic Co-operation and Development (OECD) reported that in 2004 alone, there were 3.4 million sub-Saharan Africans officially identified in EU member states [9]. Despite this, data on the health of these populations are rare [6,10]. For instance, on cardiovascular diseases only a few studies have been made among Sub-Saharan African populations in Europe [6]. In addition, many of these populations are confronted with issues of low socio-economic status, acculturation and language difficulties coupled with different cultural beliefs such as trust in traditional medicines [11-13]. Some analysts have asserted that it is only when the health needs of migrants are addressed would the host countries be able to support the health of the country as a whole [14]. Although some central governments have identified this issue as an important public health concerns, much is desired in the analysis of the policies that are implemented [15].

Knowledge about the enablers and barriers in access to healthcare experienced among migrant and ethnic minority groups is important in promoting healthcare access and, ultimately, addressing their health needs. A number of studies among migrant and ethnic minorities have revealed important barriers in healthcare access [5,16,17]. However, less is known about the enabling factors, while insight into these is equally important in generating solutions to enhance health care access. Therefore, with this study we aimed to explore the enablers as well as barriers in access to the Dutch healthcare system among the biggest sub-Saharan African population (Ghanaians) living in Amsterdam, the Netherlands.

## Methods

We conducted a qualitative study using focus group discussions. Focus groups were used in this study because it offers participants the opportunity to share their opinions and address themes that researchers may not have anticipated [18]. The study was approved by the Medical Research Ethics Committee of the Academic Medical Centre of the University of Amsterdam.

### Participants and recruitment

There are currently about 20,000 documented Ghanaians living in the country according to the Central Bureau of Statistics [19]. Ghanaians first settled in the Netherlands between late1970's and early 1980's, mostly for economic reasons. A second wave of immigration of Ghanaians occurred in the early 90's mainly for family reunion [20]. Amsterdam, particularly the southeast district became home for most Ghanaians [21]. Therefore our study was based in this district. The community is tightly knit with a number of established cultural networks including faith-based organizations, cultural groups and media groups (television and radio stations). As we aimed to include a broad representation of the community, we worked within these networks to recruit participants for the focus groups. We assumed that a community-based approach would enhance participation.

### Conduct of groups

Three of the focus groups were held at the Academic Medical Centre (AMC) and the other three at community centres where the participants usually meet. Each location was chosen by participants for their convenience. At the end of each discussion, participants received a shopping voucher to the value of 10 euros for their participation. Participants were informed about the aim of the study and they gave their verbal consent to participation. Each participant completed a short anonymous questionnaire on their demographics. They were assured of anonymity in the presentation and publishing of the data.

Discussions were chaired by a moderator and assisted by a facilitator who made notes. L.B moderated 4 groups, 2 each conducted in English and Twi (a language common in Southern Ghana where a large proportion of Ghanaians in Amsterdam originate); whilst M.N. chaired the other 2 groups in English. Members of the day's discussions introduced themselves after the moderator presented the purpose of the meeting. Following the norms of the Ghanaian cultural traditions, an ice-breaking topic was loosely discussed to enable participants relax and prepare for the main topics. These topics varied across groups; for instance the younger participants (18-30 yrs) were asked to describe whether they associated more with their father's or mother's birth town/village. The duration of the discussion varied from 90 to 140 minutes (mean duration about 100 minutes).

**Figure 1:**
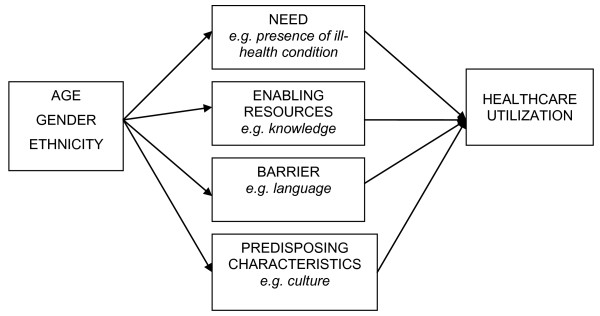
**Adapted Andersen model**.

The discussion guideline (Table 1) was semi-structured and topics were discussed exhaustively. Four main themes formed the guideline, which included perceptions on general health and well-being and access to the Dutch healthcare system. We used concepts of the Andersen's behavioural model [22] (see Figure 1). Discussion topics revealed the need, enabling factors, barriers and predisposing characteristics of Ghanaians living in Amsterdam on their perceptions and access to healthcare.

**Table 1:** Discussion guideline about perceptions in healthcare and access to healthcare

Main theme	Sub-theme
Perceptions on general health/well-being	• What are common health problems and how do you usually report them for healthcare?
	• What are your health concerns/priorities

Perceptions of the Dutch healthcare system	• Does the Dutch healthcare system meet your need(s)?
	• What would you have changed in the present healthcare system?
	• What do you particularly like about the healthcare system?

Enabling in seeking healthcare	• What factors make it easier for you in seeking healthcare?
	• What factors make it difficult for you in seeking healthcare?

Barriers to access to healthcare	• What is the usual reception (e.g. language, general atmosphere) at your healthcare facility?
	• What are your beliefs in the care (e.g. prescriptions/advice) offered by the healthcare worker at these facilities?

Discussions were recorded, transcribed literally and analysed using qualitative data software MaxQDA. Discussions conducted in Twi were translated into English by LB, who originates from Ghana. A coding framework of themes was developed based on the theoretical model. Memos were written for each code to guide the coding of texts. All transcripts were coded separately by L.B and M.N. and later compared. Samples of texts were selected for their appropriateness in describing each block in the theoretical model.

## Results

### Characteristics of study participants

Six focus groups were conducted from November 2009 to February 2010 (N = 51). Each focus group was made up of a convenience sample of 4-11 persons. Participants were mainly first-generation migrants aged 18 years and older. Table 2 gives the demographics of the participants in the focus groups. Five of the focus groups were mixed sex and one all-women's group. Among the mixed groups were an all-Muslim group and one all-Christian group. The majority of participants were born in Ghana and more than half had up to secondary school education. Less than a quarter of the fifty-one participants migrated to the Netherlands prior to 1990; whilst a third was residing in the Netherlands for < 10 years.

**Table 2:** Demographics of the study participants (*n = 51*)

Demographics	Number (%) of participants
Gender (n = 51)	

Male	22 (43.1)

Female	29 (56.9)

Country of birth	

Ghana	46 (90.2)

The Netherlands	5 (9.8)

Year of migration	

< 1990	11 (23.4)

1991-2000	22 (46.8)

2001-2009	14 (29.8)

Age	

18-30	16 (31.4)

31-40	13 (25.5)

41-50	15 (29.4)

51-60	5 (9.8)

> 60	2 (3.9)

Education level	

Primary	7 (13.7)

Secondary	28 (54.9)

University/Tertiary	16 (31.4)

Living situation	

Studying	17 (33.3)

Employed	18 (35.3)

Unemployed	11 (21.6)

Unable to work	5 (9.8)

Household situation	

Couples with children at home	24 (47.1)

Couples without children	4 (7.8)

Single parent with children at home	11 (21.6)

Living alone	7 (13.7)

Others	5 (9.8)

The youngest participants (18-30 yrs) were all university students or graduates. Eighteen of the participants were employed whilst 11 said they were unemployed. Table 3 shows descriptive information of the focus groups.

**Table 3:** Descriptive information of focus groups (*N *= 51)

Group	Location	Language used in discussion	Gender/number of participants	
			**Male**	**Female**

A1- A4 *(n = 4)*	AMC	English	2	2

B1- B5 *(n = 5)*	Community centre	Twi	4	1

C1- C9 *(n = 9)*	AMC	English	5	4

D1- D9 *(n = 9)*	AMC	Twi	0	9

E1- E13 *(n = 13)*	Community centre	English	5	8

F1- F11 *(n = 11)*	Community centre	English	4	7

Each quotation identifies the participant and the group in which he/she belonged; A, B, C correspond to groups 1, 2, 3, respectively; for example C5 represents participant number 5 in group 3 (Table 3). We used the average age of participants and the group name when quoting individuals; except for group A where participants filled out their actual ages in the short anonymous questionnaire. The following summarizes the main issues which determine the enablers and barriers in access to healthcare. There are four main blocks using the concept of the Andersen model (need, enabling factors, barriers and predisposing characteristics) of the study population.

In this study, we gathered the three central sub-codes for each block derived from the most coded texts, except for the need block which was mainly of perceived health or ill-health. Participants' opinions have been quoted verbatim, except for names which are withheld.

### Enabling factors

Enabling resources refer to those resources acquired by an individual which influence or contribute to decisions or choices in seeking healthcare [22]. Participants revealed the following sub-themes as their enabling factors in access to the Dutch healthcare system.

#### Knowledge

Knowledge about diseases common in the community appears to be an important driver for seeking healthcare. Many mentioned that high blood pressure, diabetes, stroke, cancer, fibroid, asthma, anaemia, body pains, stomach pains and depression were important issues in their community with high blood pressure being perceived as an increasingly important problem. Participants indicated that stories of ill-health and, potentially, dramatic consequences were readily circulated, contributing to overall awareness and concern within the community.

Some participants emphasized that having knowledge both about the healthcare structure and their ill-health condition enabled them to access available care. Additionally, younger participants mentioned the internet as a source of information on health conditions, and some mentioned informing themselves prior to visiting the General Practitioner (GP).

Formal education was seen as an important condition for obtaining knowledge and some expressed the idea that education enables migrants to explore their possibilities in almost everything in life.

*"When you migrate, educated people will find ways to figure things out". (Male, 31 yrs- A2)*.

#### Perceived quality of the healthcare system

Perceived quality and efficiency of the Dutch health system was one of the main enabling factors of accessing health care. The Dutch health system here refers to all aspects of healthcare such as from information from the Municipal council, the GP to specialists in hospitals. Participants described their appreciation in terms of the structure and organization of the healthcare system and contrasted this with their experiences with the system in Ghana.

*"First of all, I think the system is informative. The delivery of information is usually through the press, radio and television, which I think is good .... Secondly, I appreciate the doctor-patient ratio; I assume a doctor can only attend to a few number of people at a time. Unlike in Ghana, one doctor may be attending to about thousands of people....this I think is bad. I think the Dutch delivery system is very efficient. Lastly, when your house doctor prescribes medication for you, he knows the pharmacy will have it, so you only walk in and collect your medication. (Male, 55 yrs- B1)*.

Another participant emphasized the better sanitary conditions in Dutch hospitals; for him, that is an assurance of safety.

*"The day I came to the (.....) Hospital, the theatre was very clean. I said to myself that oh today, here I know I'm not going to die. Because in Ghana when you are going to the theatre, everyone is like hey, he is going to die". (Male, 41 yrs- F3)*.

#### Community initiatives/cohesion

Many Ghanaians appreciate community support as a place for enlightenment on many issues including health. Community organizations in southeast Amsterdam occasionally organize programmes to educate and inform the community on health. This way the community serves to enable those individuals with limited formal education or understanding of the healthcare system in accessing healthcare.

*"I know there are a lot of different Ghanaian organisations .... Through discussions, you can really find out about that generation and indeed the views towards the hospitals or the other way round, we can work towards solutions". (Male, 31 yrs- A2)*.

#### Availability/accessibility of family support

Family systems are important part of the Ghanaian culture; members are dependent on each other for various kinds of support, including accompanying various family members to the doctor in order to offer support or to act as a translator. One young man accounted the importance of his support to his mother:

Moderator: And how do you find it? Like going to the doctor with your parents? It can't be easy, isn't it?

*"I have no problem with that. Because they are my parents and I don't have to think twice to do it, so I do it" (Male, 22 yrs- A4)*.

### Barriers

Barriers are described as resources both personal and societal that are constraints or tend to hinder efforts in seeking healthcare [22]. Here, participants revealed that communication or expression and language, trust in the doctor and perceived benefit or value of the healthcare system as barriers.

#### Communication/expression and language

Approximately a third of participants mentioned not being able to speak the Dutch language or to express themselves as one of their major barriers in accessing healthcare. In addition, it was often mentioned that communicating in English was not always an easier option.

*"English is not really our language. And even for those who have attended school in Ghana, some find it difficult to express themselves. Besides, [English] it is not an easy language too. Even for the Dutch, some find it difficult to express themselves or communicate how they feel to their doctors. So, language should not be underestimated". (Male, 25 yrs- C5)*.

There were different opinions about use of language (English/Dutch) and openness to communication using alternative means. A few participants thought that an individual's ability in expression would achieve the needed attention from his/her audience.

*"I think it's all or a part of communication and also non-verbally how you portray yourself. Because even if it's broken English, you can express yourself a little bit. And you can make clear signs with your hands and feet or whatever; the other person doesn't have a choice but to listen to you". (Female, 33 yrs -A1)*.

#### Trust in doctor/system

The issue of trust was hotly discussed in all focus groups with many expressing a deep distrust of doctors and the system as a whole.

Firstly, the matter about prescriptions was greatly debated by all groups. Whilst some resented what they saw as a reluctance of doctors to prescribe medication, others were wary of the drugs they received and thought they were 'test prescriptions'.

*"And when it comes to the time when they have to prescribe something for you; they behave as if the drug they are prescribing, they are going to test it on you. You can get the feeling that he [the doctor] is trying you like an experiment". (Male, 45 yrs- F6)*.

Secondly, participants expressed dissatisfaction with the level of medical expertise and the lack of continuity of healthcare received, particularly in hospital settings. Being attended to by junior doctors engendered a lack of confidence in the treatment received; a feeling that was exacerbated during follow-up visits where participants reported being faced with yet another junior doctor.

*"One more thing that I don't really like about the system; you are attended to by different doctors. Anytime you go to say the [local academic hospital]; you don't meet the one who you first met. That is confusing, because then they don't know you or take time to study your file". (Female, 45 yrs- F4)*.

Thirdly, many were sceptical about the way healthcare insurers handled migrant clients. Some participants expressed the belief that healthcare insurance companies are not transparent to them since they (migrants) lack an understanding of the health insurance system and the way levies are calculated. A common perception related to frequent changes in the organisation of health insurance, causing confusion and undermining the rights of the patient. In addition, many participants expressed suspicion of collaboration between insurance companies and GPs, pharmacies and hospitals. One man suspected that the insurance companies dictate to hospitals and doctors.

*"Yes, it goes wrong when it comes to spending. The hospitals make their monies from the insurance companies. Some of the hospitals allow the insurance companies to dictate to them. But the doctors should know that they have sworn to save lives first". (Male, 45 yrs- F5)*.

#### Perceived benefit/value of the healthcare system

Although, the appreciation of a quality and efficient system facilitated access, here many participants revealed distaste for medical care in any form other than prescriptions. They place more value on medications than advice.

*"Yes, quite recently, I had pain in my left leg and my house doctor referred me to a specialist in a hospital. The specialist didn't do much with me, except examining my leg and pressing it here and there. I was not given any kind of medication, only an advice." (Female, 41 yrs- D9)*.

This was a general perception expressed across all groups of participants. One group debated this issue extensively; one member being a medical student with knowledge about prescription protocol tried to defend the medics.

*"But there is this protocol that I know; they start with the cheapest drug first. So when you visit your GP, he will not prescribe say the 100 euro drug first, maybe the 50/60 euro..." (Male, 25 yrs- C7)*.

*"So they don't prescribe the drugs that will cost the health system?" (Female, 25 yrs- C8)*.

*"But listen.....if we all want a medicine of 100 euro, can you calculate how much it will cost the healthcare system?" (Male, 25 yrs- C7)*.

*"But you have to understand that the healthcare is not free and we know that. It is not like we don't pay anything and all of a sudden we want some expensive medicine. I pay over 100 euro a month, therefore when I am sick; I expect to get the best of care". (Male, 26 yrs- C6)*.

#### Expectations regarding treatment

Expectations regarding treatment were similar across the different groups. In Ghana, doctors routinely examine the patient such as checking their pulse, temperature and blood pressure during every consultation. This is in contrast to practice in the Netherlands, where the general practitioner (GP) will often rely on a description of the patient's symptoms in making an initial diagnosis. However, some participants mentioned that many Ghanaian migrants are not used to explaining their symptoms to their GP. The expectation among many is that the GP should be able to make a diagnosis based on a physical examination. The inadequacy of language to describe symptoms as well as differences in expectations regarding the doctor's actions was identified as a problem.

Additionally, participants felt that Dutch doctors are less emphatic, which was exemplified by the fact that they spend less time with their patients.

*"When I went to my house doctor last month with pain in my breast and chest area, I was expecting her to at least come over and examine me; instead she used that thing....stethoscope to check my heartbeat and then she said everything is alright." (Female, 26 yrs- C1)*.

The issue with time spent at the GP's was different for most participants, even though averagely many were dissatisfied with it.

*"Eh, the longest time I've had... that I can say this doctor has read something about me is 5 min." (Male, 45 yrs- F5)*.

However, some were consoled with the perception that this was due to the system as a whole which only allows a minimum time with patients.

*"But also it's the system because the house doctor gets like maybe 10 min per patient but he has in his whole case log maybe about 500 people". (Female, 33 yrs- A1)*.

Although our primary aim was exclusive of the enablers and barriers in access to healthcare, we encountered some underlying issues that came along with them. Thus, the following themes were included to understand clearly the issues in enabling factors and barriers.

#### Culture and acculturation

The attitude of an individual as part of their culture was mostly mentioned to be related to their level of acculturation. Some admitted that the Ghanaian culture sometimes influenced their perceptions in access to the Dutch healthcare system. Participants described their perception of the Dutch culture and compared with the Ghanaian culture.

*"That is why I wanted to say that culture plays a big role here. Here in this country, we talk about everything...the Dutch way of doing things; they talk about everything from A to Z. But we [Ghanaians] have hyphens and clauses and codes". (Female, 45 yrs- F5)*.

The influence of acculturation centred mostly on the factors that the participants themselves perceived as indicative of acculturation level. Command of the Dutch language was cited as not only an important measure but also a necessity. In combination with sufficient education, this was seen as an essential condition for independence within the Dutch society; having the ability to function with less or no kind of assistance.

*"So I think for our generation, if you go to school and are educated [and speak Dutch], you should be able to do things on your own. You don't necessarily need other people. From the simple thing I remember from a long time, translating letters should be on your own". (Male, 22 yrs- A2)*.

#### The role of stress

Participants discussed their perceptions in health in general including not only physical health but also mental well-being. Many believe that stress could be the underlying cause of most of their ill-health conditions. Stress was often seen to be acquired as a result of migration and their living situation in the Netherlands; the demands of their daily routine as well as financial pressures. The assumption was that the ill-health condition would be cured when stress is treated.

*"Most of the people have high blood pressure according to the situation they are living here. The government is giving you money pressure; lots of people have debts.... Lots of people have so much to think and with that you can get high blood pressure". (Male, 45 yrs- F4)*.

Furthermore, some perceived they are healthier during holidays in Ghana. One participant attributed this to the weather in Ghana and expounded that a prior medical preparation for a holiday in Ghana may sometimes be unnecessary.

## Discussion

### Key findings

In this study, we explored the enabling factors and barriers in access to the Dutch healthcare system among Ghanaians in Amsterdam. Knowledge and the perception of an efficient and quality Dutch healthcare system have been revealed as facilitators in access to healthcare. The system is said to be informative and has an impressive doctor-patient ratio. Perception of greater benefits from the Dutch healthcare system compared to Ghana overlay cited barriers. Such perceptions may augment our estimation that, majority of participants who migrated prior to year 2000 may have carried with them their experiences of the healthcare delivery in Ghana. Appreciation of better facilities in all levels of healthcare from the physician's office to specialists in the hospitals enabled and also motivated access. Awareness of diseases common to the group such as high blood pressure, diabetes, stroke, cancer and depression are widespread in the community. Such awareness generates desire for knowledge about these diseases and the need for care. Community cohesion and initiatives provide some enlightenment, including health information for members.

### Limitations and strengths of the study

One of the limitations of this study is the selection and grouping of participants. We asked key persons in the community to assist in recruiting participants since they have contact with a cross section of adults aged 18 years and older. This approach was successful in that it worked within established networks, in a culturally sensitive way. However, we could not influence sampling because it was not feasible to set specific criteria for recruitment despite emphasis on age, gender and socio-economic status. Furthermore, only participants who speak and understand Twi and/or English were invited due to constraints in translation logistics. However, although not the first research among Ghanaians in Amsterdam [12], this is the first qualitative research that explored the facilitators and barriers in access to the Dutch healthcare system from a broad sample perspective. The specific contribution of the present study is that it investigated in detail, the sources of the enablers and barriers for the Ghanaian group in their access to the Dutch healthcare system. Another strength of the study can be derived from the mix of participating members; Christians, Moslems, first and second generation, young, middle-aged and old, men and women, low and high educated. The diversity of the participants contributes to the potential for transferability of the findings to other Ghanaian migrant communities.

### Discussion of key findings

#### Comparison with existing literature

This study expounded that the living and working conditions of many Ghanaians generate stress which many participants viewed as a causal factor for most of their ill-health conditions. The issue of stress as an underlying factor for the decline in their health status confirms similar findings from Dean & Wilson (2010) and Beune *et al. *(2006). In our study, participants described their main stressors comprising of issues with immigration status, living and working conditions and family obligations to members especially in Ghana. However, a study by Knipscheer & Kleber (2007) found Ghanaian migrants are able to cope with challenges related to migration and acculturation in a flexible way and maintain health.

As already known, many non-western ethnic minorities have low socio-economic status [23,24]. The results of our study resemble those from previous studies among other non-western migrant groups in the Netherlands, for example, Turkish and Moroccans. In spite of this, we found that Ghanaian migrants in the Netherlands, are quite highly educated (> 50% of participants educated to secondary level) and we propose this may contribute to better stress management. We estimated that being educated enables them to cope with these stressors in a relatively 'healthy' way; although dealing with stress may not be the solution in some situations. Moreover, the perception that stress is a major underlying cause of ill-health may also be a barrier for seeking appropriate or immediate healthcare and, potentially, be a barrier to treatment compliance [12].

Whilst predisposing characteristics may be inherent and peculiar to a group of people, differences occur among individuals. The culture of any group of people is said to function in the lives of the individuals who compose them [25]. It can be said that culture appears to influence and explain how some Ghanaians access healthcare. The availability of family and or social support as well as community initiatives added to the list of enablers. Traditionally, Ghanaians uphold family and or social support systems and some depend on such values from relatives in their access to healthcare [20,26]. Even though our study did not investigate such support; some of the younger participants revealed support to their parents' healthcare utilization.

Ghanaians have been living in the Netherlands for quite some time so one would expect a reasonable level of acculturation and understanding of the health system. However, we did not find this in our study. Therefore, our finding reflects poor acculturation level, particularly on the issue of language (which was a focus among the groups when they talked about acculturation). In addition, many Ghanaians in Amsterdam live in a "non-Dutch" environment; Amsterdam south-east is a district with many ethnic minorities, including Surinamese and other African groups which may impede acculturation into mainstream Dutch society. In order to promote acculturation among Ghanaians and similar groups such as other sub-Saharan Africans, interventions are needed that suit their specific needs and consider their experiences to moderately incorporate them in the host country [11,26].

In spite of the aforementioned enablers, difficulties with the Dutch language influenced access to healthcare for many Ghanaians. This is consistent with findings from a study by Denktas and colleagues (2009) in which Dutch language competence was high in explaining ethnic differences in health services utilization. In Norway, language difficulties were an explanation for the lower satisfaction with physicians and participation in health surveys among non-Western immigrants [5]. Despite difficulties with the Dutch language, participants revealed that most Dutch doctors are able to communicate in English, especially those in Amsterdam Southeast because they are used to patients originating from English-speaking countries. Communication with other groups like the Turkish and Moroccans is a larger problem. We expected that English language would not be as challenging as the Dutch for many Ghanaians since Ghana was a former British colony and thus many are conversant with the English language. Besides, studies among Ghanaians in English-speaking countries like in the UK and US did not report language difficulties at least as a barrier in access to healthcare [16,17]. Contrary to expectations, many participants in this study indicated that their command of English was not sufficient to enable them to adequately communicate with healthcare providers, despite the relatively high level of education among our respondents. Recently, a study by Priebe and colleagues (2011) gathered that, healthcare professionals across several European countries admitted language barrier is one of the problems in providing quality of care to migrants [27]. Therefore, our finding is important for policy and service delivery as most of the GPs expect Ghanaians to be able to cope in English.

Lower levels of trust in physicians and the healthcare system are common among many ethnic minorities. Some reiterated that lower levels of trust are mostly derived from language difficulties [5,28]. In the Netherlands, Suurmond and colleagues found one of the situations for low trust in the physician was inadequate exchange of information between the migrant patient and the healthcare provider [29]. Moreover, healthcare professionals from several other European countries acknowledged differences in the understanding of illness and treatment with migrant patients [28]. Our study elucidated low levels of trust in physicians and the healthcare system on the subject of prescriptions, advice and continuity of care, as well as care provider's prejudicial behavior which many perceived as apathy. It can be said that the attitude of some doctors are less of emphatic and more of the effect of their training or the structure of the healthcare system.

Nonetheless, we found some explanations for low levels of trust exclusive of other studies. Among them was a lack of transparency in the deduction of health insurance levies. The privatization of the Dutch health insurance is not implicitly appreciated by many Ghanaians, some of whom migrated with their experiences of the so-called "out-of-pocket" healthcare delivery in Ghana. Interestingly, even present day healthcare in Ghana is delivered via a health insurance system [30]. Many participants have little or no insight in the operations of a health insurance system. However, after about three decades of residence for the first few migrants, integration remains a challenge to many of such Ghanaians [31].

Besides the aforementioned barriers, this study revealed that the Ghanaian group appreciates other aspects of the Dutch healthcare system, such as the doctor-patient ratio. As a matter of fact, culture-specific education and attention for communication skills may be novel in the medical training of most European countries [32-34]. In spite of this, we anticipate that initiatives to improve interpersonal skills for healthcare providers would help lower distrust for this migrant group.

#### Implications for policy and service delivery

Although our findings may add to current literature on challenges in access to healthcare for migrant and ethnic minority groups such as Ghanaians in the Netherlands, the enablers revealed are worth noting. With regard to the language barrier, communicating in Dutch is a huge problem for most Ghanaians. Moreover, their limited command of English does not enable adequate communication with healthcare providers who are willing to use this language. The latter finding is particularly relevant. Many Ghanaians can "get by" with English and therefore many health care providers are unaware that there is a language barrier when communicating with this group. As a result, the use of available professional interpreting services is underutilised.

Although knowledge about the Dutch healthcare system facilitated access, insufficient insight in the operations of the health insurance system generates distrust. In the Netherlands, ethnic healthcare advisors are used as strategy to bridge the gap between ethnic minorities and healthcare services [35]. However, since community cohesion and initiatives are platforms for enlightenment among our study group, it is important for policy makers in the planning and disseminating of information to consider such platforms. For instance, health insurance companies may promote knowledge about their operations through community initiatives organized by Ghanaians.

On the issue of low trust, healthcare providers would benefit from knowledge of the healthcare system of migrants' country of origin and the way their previous experiences may influence their expectations in the healthcare delivery in the Netherlands. Secondly, healthcare providers can build trust by being transparent about the decisions underlying choice of treatment. Finally, it seems important to engender trust simply by employing a more hands-on approach during medical examinations and by being more explicit in explaining why a physical examination may not be required.

## Conclusions & recommendations

Knowledge and the perception of an efficient and quality Dutch healthcare system enabled access to healthcare. Community initiatives/cohesion and the availability/accessibility of family support added to the enabling factors for Ghanaians in Amsterdam. However, poor communication in the Dutch language, as well as in English and mistrust in health care providers and the healthcare system appear to be important barriers in accessing healthcare. Experiences from the healthcare system in Ghana reflected in perceptions of the Dutch healthcare system. The enabling factors identified are important for promoting healthcare access among this and similar Sub-Saharan African communities.

## Competing interests

The authors declare that they have no competing interests.

## Authors' contributions

KS and CA conceived the project. LB conducted 4 and MN conducted 2 of the focus group discussions. LB and MN analysed and interpreted the data and LB took the lead in writing the article with input from MN. HD, KS and CA contributed to the interpretation of the data and the preparation of the article. All authors read and approved the final manuscript.

## Ethical approval

The Medical Ethics Committee of the Academic Medical Centre, University of Amsterdam approved the study.

## Pre-publication history

The pre-publication history for this paper can be accessed here:

http://www.biomedcentral.com/1472-6963/12/75/prepub
